# Cold Feet

**Published:** 1879-06

**Authors:** 


### COLD FEET

Often produce a burning sensation in the throat, which if allowed to continue in operation, ultimately undermines the health; the reason is, less blood being in the feet than is natural, there is an extra amount at the other end of the body; can any thing be more absurd than to clip off a man's palate, whack out his tonsils, and "burn out his throat," for such an ailment; can that send warmth to the feet? can we purify the fountain, by purifying the stream? When will men learn to think for themselves?

My experience is, *Throat-Ail is not to be radically and permanently cured in any case, except by rectifying first, and then building up the general health of the system*, and that requires time, determination, and systematic habits of rational life. Who thinks differently, and acts up to his belief, will find himself just as miserably deceived, as that unfortunate class of theologians, who assert "It is no matter what a man believes, if he is sincere in his belief." Is not such a logician a "sincere" fool? *Clergyman's Sore Throat is better cured*, AS A GENERAL RULE, *in the continuation of ministerial duty.* My ordinary advice is, PREACH EVERY DAY AND SUNDAY TOO, rather than once a week. These fitful efforts are often a main cause of Throat-Ail; just as a man who travels ten miles a foot on Sunday, and on other days none at all, will be wearied every Sunday night; whereas, were he to walk five or six or eight miles every day, rain or shine, he would perform ten or twelve on the Sabbath, without appreciable fatigue. Men of "The Cloth," why don't you think for yourselves? Sometimes I think I am not altogether a drone in creation, because there are excellent men now, in different parts of the country, whom I have never seen, who, having abandoned preaching, applied to me for advice, and on being urged to resume pastoral charges immediately, as a means of cure, have done so, and have steadily recovered, and are now bearing "the burden and heat of the day." So that I am every Sabbath preaching by proxy, to many a listening multitude. It is not politic to say here how many I have killed off, or to inquire if those referred to might not have recovered without doing any thing. They came and were cured as antecedent and sequent, not necessarily as cause and effect.

